# Selective Eradication of Colon Cancer Cells Harboring PI3K and/or MAPK Pathway Mutations in 3D Culture by Combined PI3K/AKT/mTOR Pathway and MEK Inhibition

**DOI:** 10.3390/ijms24021668

**Published:** 2023-01-14

**Authors:** Velina S. Atanasova, Angelika Riedl, Marcus Strobl, Julia Flandorfer, Daniela Unterleuthner, Claudia Weindorfer, Patrick Neuhold, Simone Stang, Markus Hengstschläger, Michael Bergmann, Helmut Dolznig

**Affiliations:** 1 Institute of Medical Genetics, Medical University of Vienna, Währinger Straße 10, 1090 Vienna, Austria; 2 Department of General Surgery, Division of Visceral Surgery, Medical University of Vienna, Währinger Gürtel 18-20, 1090 Vienna, Austria; 3 Members of the Comprehensive Cancer Center Vienna, Medical University of Vienna, 1090 Vienna, Austria

**Keywords:** 3D culture, targeted therapy, colorectal cancer, organoids, precision medicine

## Abstract

Colorectal cancer (CRC) is the second deadliest cancer in the world. Besides APC and p53 alterations, the PI3K/AKT/MTOR and MAPK pathway are most commonly mutated in CRC. So far, no treatment options targeting these pathways are available in routine clinics for CRC patients. We systematically analyzed the response of CRC cells to the combination of small molecular inhibitors targeting the PI3K and MAPK pathways. We used CRC cells in 2D, 3D spheroid, collagen gel cultures and freshly isolated organoids for drug response studies. Readout for drug response was spheroid or organoid growth, spheroid outgrowth, metabolic activity, Western blotting and immunofluorescence. We found profound tumor cell destruction under treatment with a combination of Torin 1 (inhibiting mTOR), MK2206 (targeting AKT) and selumetinib (inhibiting MEK) in 3D but not in 2D. Induction of cell death was due to apoptosis. Western blot analysis revealed efficient drug action. Gedatolisib, a dual PI3K/mTOR inhibitor, could replace Torin1/MK2206 with similar efficiency. The presence of PI3K and/or RAS-RAF-MAPK pathway mutations accounted for treatment responsiveness. Here, we identified a novel, efficient therapy, which induced proliferation stop and tumor cell destruction in vitro based on the genetic background. These preclinical findings show promise to further test this combi-treatment in vivo in mice and to potentially develop a mutation specific targeted therapy for CRC patients.

## 1. Introduction

According to GLOBOCAN 2020, adenocarcinoma of the colon and rectum (colorectal cancer, CRC) is ranked as the third most common cancer in men (600,896 cases, 302,117 deaths) and the second in women (547,619 cases, 274,7410 deaths) in the world. In early stage localized carcinomas a 5-year relative survival rate of 90.3% in CRC patients in the United States was reported [1]. This is due to the fact that surgical removal of the tumor is most efficient at this time period. However, only 40% of CRC are detected at this early stage. In case of advanced CRC, e.g., when the cancer has spread to distant organs, the 5-year survival rate drops to 12.5%. Treatment options for patients with advanced colon cancer include (neo)adjuvant chemotherapy (oxaliplatin, irinotecan, 5-FU). Finally, in metastatic CRC, various drugs are used either alone or in combination, including chemotherapy, EGFR and VEGFR (e.g., bevazizumab) targeting, and multikinase targeting (regorafenib) as third-line treatment [2]. However, relapse rates are high for advanced CRC due to the development of resistance mechanisms to chemo- or targeted therapies [3].

PI3K pathway and MAPK pathway mutations are frequently mutated in CRC and promote tumor development in concert with other common mutations of the WNT signaling, p53 and TGFb signaling pathways [4]. In fact, in non-hypermutated tumors (which are 90% of all CRC) about 59% of all metastatic CRC patients harbor mutations in the MAPK pathway (KRAS 44%, BRAF 8.5%, NRAS 3.9%, MAP2K4 1.7% and MAP2K1 0.8%). A total of 26.7% of these CRC patients have mutations in the PI3K/AKT/MTOR pathway (PIK3CA 18%, PTEN 2.8%, PIK3CG 2.5%, PIK3R1 2.4% and AKT1 1%) [5]. Moreover, another 11% show mutations in receptor tyrosine kinases, being upstream of both pathways [5]. Similar numbers at slightly reduced frequency are found if stage I-IV tumors are analyzed [4]. A high proportion of mutations in the same pathway are mutually exclusive. Thus, combinatorial target inhibition of these pathways presents a promising strategy to treat the majority of CRC patients with widely available kinase inhibitors and to overcome potential resistance mechanisms, which arise from single target treatment as shown for the MEK inhibitor pathway [6]. So far, no treatment options targeting these pathways are available in routine clinic for CRC patients. However, there might even be an additional benefit for patients treated with combinatorial MEK and PI3K inhibition as it not only inhibited tumor growth but also counteracted cancer cachexia in mice [7].

3D tumor cell cultures better mimic solid human tumors than cancer cells cultured as monolayers on plastic [8,9,10]. In multicellular spheroid 3D models gene expression [11], response to treatment [12,13] and PI3K/AKT/MTOR signaling [14], to name a few, resemble more closely the in vivo situation. Therefore, 3D cancer models are now well accepted to be biologically relevant for drug development and preclinical drug testing [15,16,17]. Even more intriguing, patient-derived cancer organoids not only grow in 3D in extracellular matrix but also closely recapitulate patient diversity in respect to tumor architecture and mutational landscapes [18]. Thus, these sophisticated models are ideally suited to validate findings made in conventional cell lines and translate them to a personalized setting with a superior physiological relevance [19].

The objective of this study was to systematically analyze the response of CRC cells and organoids to the combination of small molecular inhibitors targeting important hubs in the PI3K and MAPK pathways in 2D and 3D. We aimed to focus on identification of combinations of inhibitors, which induce profound tumor cell destruction preferentially in the 3D experimental setup. We also used gedatolisib (PKI-587), a dual pan PI3K and mTOR inhibitor, with promising clinical potential, which was granted fast track and breakthrough therapy designation from the FDA in 2022 and assessed its potential to replace the experimental drugs Torin1 and MK2206. Indeed, combined PKI-587 and AZD treatment of primary patient-derived colon cancer organoids was similarly efficient as T/MK/AZD treatment and associated with increased apoptosis and decreased proliferation. Interestingly, the response was only seen in cell lines or organoids harboring mutations in the respective pathways.

## 2. Results

### 2.1. Effect of Drug Combinations in 2D and 3D Cell Culture

**Figure 1:**
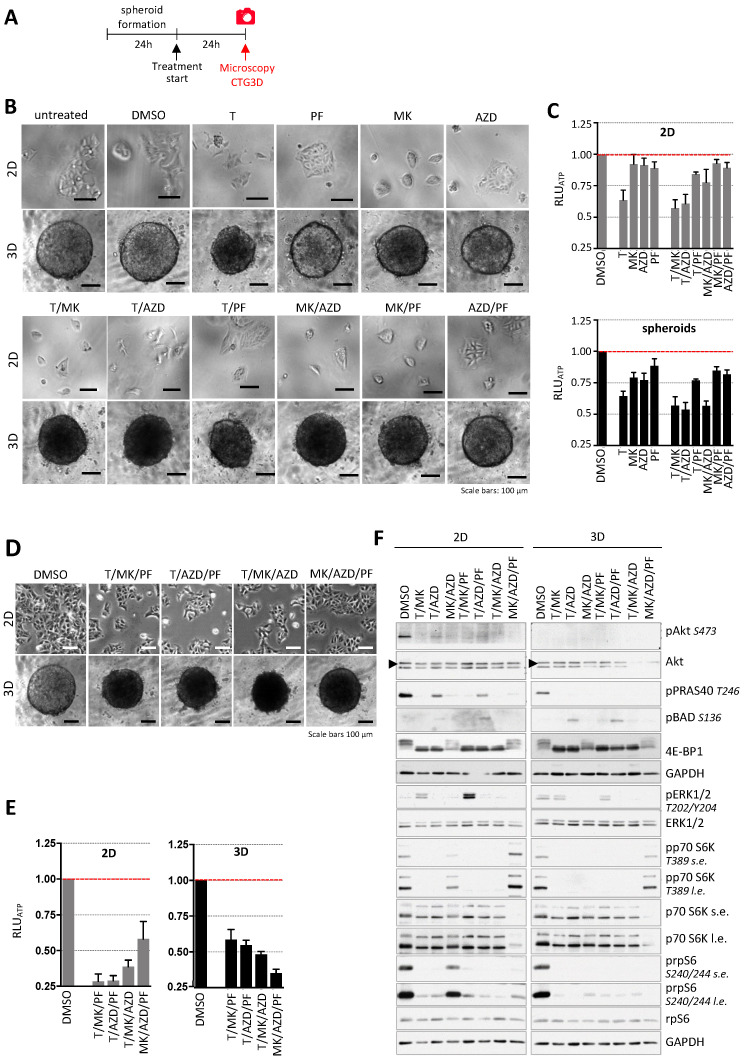
Effect of inhibitor combinations on DLD-1 cells. (**A**) Overview of the time frame of the experiment. (**B**) Representative phase contrast microscopic images of DLD-1 cells in 2D and 3D culture treated with single inhibitors and combinations of Torin1 (T, 250 nM), PF4708671 (PF, 10 µM), MK2206 (MK, 1 µM) and AZD6244 (AZD, 1 µM) for 24 h. Untreated and DMSO cultures are controls. Scale bars represent 100 µm. (**C**) Cellular ATP levels were determined with CellTiter-Glo 3D. Luminescence values (RLU_ATP_) were normalized to the DMSO controls (*n* = 6 per condition). Bar graphs are presented as mean ± standard deviation (SD). 2D in gray bars; spheroids in black bars. (**D**) Representative phase contrast microscopic images of DLD-1 cells in 2D and 3D culture upon inhibitor treatment (inhibitor concentrations as in (**B**,**C**) for 24 h. Scale bars represent 100 µm. (**E**) ATP-levels (RLU_ATP_ from CTG3D) after 24 h treatment were normalized to controls (2D: *n* = 6 and 3D: *n* = 9 per condition). Bar graphs are presented as mean ± standard deviation (SD). (**F**) DLD-1 colon cancer cells were treated with a dual and triple inhibitor combinations of T (250 nM), PF (10 µM), MK (1 µM) and AZD (1 µM) for 24 h. DMSO served as control. The impact on the Akt/mTOR and Ras/Raf/MEK/ERK signaling pathways after 24 h was determined by Western blot analysis. GAPDH served as a loading control. In case of the simultaneous presence of very weak and very strong bands, short exposures (s.e.) and long exposures (l.e.) are shown.

In a first set of experiments, we evaluated the impact of several small molecule inhibitors targeting major signaling hubs of the PI3K/AKT/mTOR and the Ras/RAF/MAPK pathway in DLD-1 colon cancer cells in conventional 2D (3000 cells/well) and 3D spheroid (3000 cells per spheroid) cultures. Cells grown in 2D and spheroids were treated with single and dual inhibitor combinations of the MTOR inhibitor Torin1 (250 nM, T), the AKT inhibitor MK2206 (1 µM, MK), the S6 kinase inhibitor PF4708671 (10 µM, PF) and the MEK inhibitor AZD6244 (1 µM, AZD, selumetinib) for 24 h (Figure 1A). Phase contrast microscopy (Figure 1B) showed that T alone or in combination with PF, MK resulted in spheroids reduced volume (Supplementary Materials Figure S1A) and with dark appearance and almost 50% reduction in metabolic capacity, determined by measuring the cellular ATP-levels compared to the DMSO controls (Figure 1C). Interestingly the treatments in 2D did not severely affect cell morphology.

Based on the effects of dual inhibitor combinations on DLD-1 cells in 2D and 3D, T/MK, T/AZ and MK/AZD were selected for further evaluation using triple inhibitor combinations. Consequently, DLD-1 cells in 2D and 3D culture were incubated with triple inhibitor combinations of T, PF, MK and AZD for 24 h. We anticipated a further reduction of cell numbers in 2D and seeded twice the number of cells compared to the experiment shown in Figure 1B,C to avoid too less cells/frame to show in representative pictures. Cells and spheroids were analyzed for spheroid size (Supplementary Materials Figure S1B), morphological and phenotypical changes and molecular differences in the Akt/mTOR and Ras/Raf/MEK/ERK signaling pathways were evaluated. Analysis of cell or spheroid morphology showed that triple inhibitor treatment of cells grossly changed neither cell morphology in 2D nor the appearance/darkness of spheroids compared to the most efficient double combination of T/MK (Figure 1D). Upon T/MK/PF or T/MK/AZD treatment, ATP levels further decreased to about 20–40% in the spheroids (Figure 1E), resulting in the most efficient inhibition. In contrast, in 2D, compared to T/MK/PF and T/AZD/PF, treatment with T/MK/AZD and MK/AZD/PF displayed less reduction of cellular ATP, clearly indicating different responses in 2D versus 3D (Figure 1E).

### 2.2. Western Blot Analysis of Relevant Signaling Molecules Revealed Targeted Drug Action

Immunoblots of different proteins of the Akt/mTOR and MEK/ERK pathways were used to assess proper drug action, as already previously established [14], and to determine molecular differences in DLD-1 colon cancer cells grown in 2D and 3D culture upon treatment with selected dual and triple inhibitor combinations (Figure 1F and Figure S1C). The presence of T in either dual- or triple-inhibitor combination treatment led to a reduction of phospho-S6 S240/244 levels in 2D which was even greater when DLD-1 cells were cultured in 3D. Furthermore, S6 phosphorylation at S240/244 was strongly decreased upon inhibition of MK and AZD in 3D compared to the DMSO control, which was less pronounced in 2D culture.

The addition of T or PF to MK/AZD treatment led to a further reduction of phospho-S6 S240/244 signals in 2D and 3D. A similar pattern was seen in protein levels of phospho-p70 S6K T389, where the presence of Torin1 led to a loss of the signal in DLD-1 cells grown in 2D and in 3D. However, whereas inhibition of Akt and MEK1 led to a slight reduction of p70 S6K phosphorylation at T389 in 2D, protein levels of spheroids were strongly decreased compared to the DMSO control. In 2D and 3D, MK/AZD/PF treatment exhibited a similar level of p70 S6K phosphorylation at T389 compared to the respective DMSO controls.

Compared to the controls, treatment of DLD-1 cells with T/MK or in combination with PF4708671 revealed an increase in p44/42 ERK1/2 phosphorylation at T202/Y204 in 2D, which was not detected in 3D. In 2D as well as in 3D, hyperphosphorylated 4E-BP1 was absent if Torin1 was present in the inhibitor combinations. However, the combination of both compounds either alone or with PF4708671 led to a slight decrease in the phosphorylation status of 4E-BP1, as demonstrated by the phosphorylation dependent mobility changes in 4E-BP1 [20], which was more profoundly seen in 3D culture. Generally, Akt phosphorylation at S473 was lost upon Akt inhibition. Torin1 treatment alone led to a strong reduction of phospho-Akt S473 levels in 2D, which were weaker when AZD6244 or PF4708671 were combined with Torin1 in a dual or a triple inhibitor combination. The same response of DLD-1 cells was seen in protein levels of PRAS40 phosphorylated at T246. Phospho-Akt Ser473 and phospho-PRAS40 T246 were not detected in inhibitor-treated spheroids (Figure 1F). Phospho-BAD levels were assessed as an independent target of AKT activity, but displayed a pattern different to phospho-PRAS40.

### 2.3. Induction of Cell Death Due to Apoptosis Selectively in the 3D Colon Cancer Model

**Figure 2:**
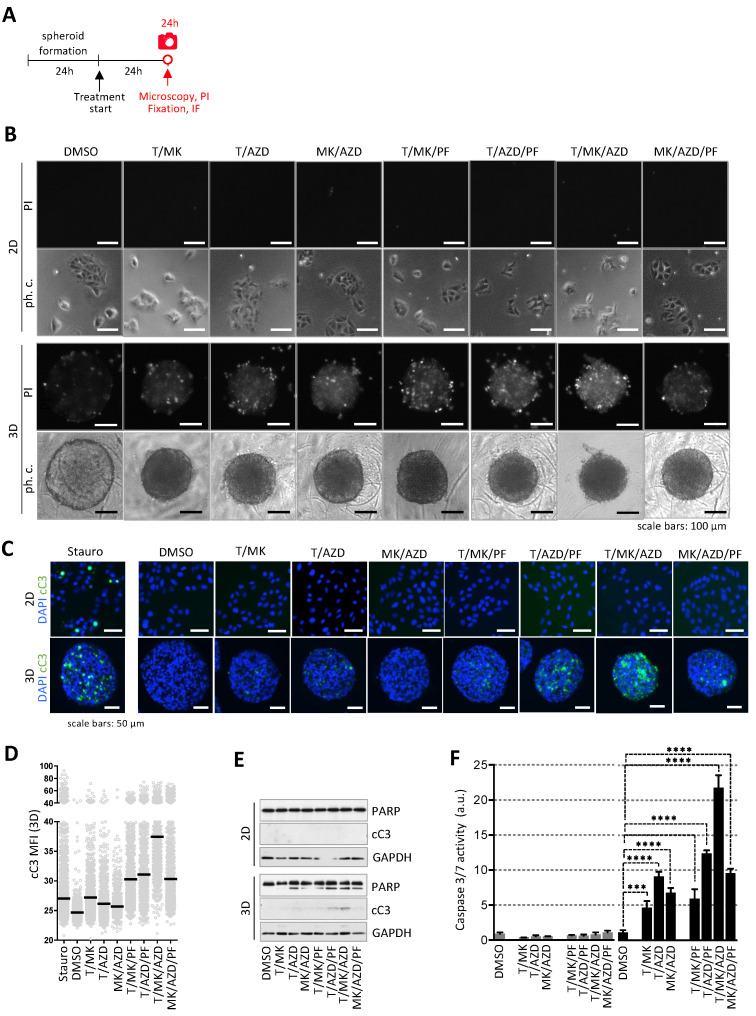
Assessment of cell death upon treatment. (**A**) Overview of the time frame of the experiment. (**B**) DLD-1 cells were incubated with dual and triple inhibitor combinations of T (250 nM), PF (10 µM), MK (1 µM) and AZD (1 µM) for 24 h. DMSO served as control. Subsequently, cells in 2D and 3D were stained with 2 µg/mL propidium iodide (PI) staining dead cells. Representative phase contrast and fluorescence images are shown. Scale bars represent 100 µm. (**C**) Cells and spheroids were treated as in (**A**), fixed and subjected to immunofluorescence analysis for cleaved caspase 3 (cC3, Asp175, green) as a marker for apoptosis. Nuclei are stained with DAPI (blue). Staurosporine (1 µM) was used as positive control. Representative images are shown. Scale bars represent 50 µm. (**D**) cC3 fluorescence intensity per cell of images from the 3D cultures (10 sections per condition) was measured in ImageJ using DAPI for segmentation. Each grey dot represents one cell, mean fluorescence intensities are indicated (black line). (**E**) Control and treated DLD-1 cells and spheroids (as in **B**) were lysed and immunoblots of PARP and cleaved caspase-3 were performed. GAPDH served as loading control. (**F**) Caspase-3 and -7 activity was determined by the ApoOne fluorescence assay (Promega, Madison, WI, USA). Fluorescence levels of treated cells and spheroids were normalized to DMSO controls (*n* = 3 per condition). Bar graphs are presented as mean ± standard deviation (SD). *p* values are indicated by asterisks.

Next, propidium iodide (PI) staining was performed to identify dead or dying cells in the cultures in 2D and 3D treated with the dual- and triple-inhibitor combinations for 24 h (Figure 2A). DLD-1 cells in 2D were negative for PI (Figure 2B, upper panels). However, double- and triple-inhibitor treatment substantially increased the number of PI-positive cells in 3D, being highest in T/MK/AZD treatment (Figure 2B, lower panels).

Additionally, in order to demonstrate that triple-inhibitor treatment such as T/MK/AZD profoundly increased the rate of cell death selectively in the 3D spheroids, immunofluorescence staining of cleaved caspase-3 (cC3, Asp175) was performed. DLD-1 cells in 2D were cC3 negative except for staurosporine treated cells, which served as a positive control. Spheroids were embedded into paraffin and stained sections revealed that triple inhibitor treatment substantially increased the number of cC3 positive cells solely in 3D with the highest levels of apoptosis in T/MK/AZD treated spheroids (Figure 2C). This was confirmed by quantitative image analysis of individual cellular cC3 levels in multiple spheroid sections of eight different spheroids using DAPI-based segmentation (Figure 2D). Further, Western blot analysis showed that the protein levels of cC3 and increased PARP cleavage correlated with the PI and cC3 staining (Figure 2E and Figure S2). Low apoptosis in DLD-1 cells cultured in 2D was confirmed by Western blot analysis, where no PARP cleavage and no activated caspase-3 were detected (Figure 2E). Determining caspase 3/7 activity by the ApoOne Caspase3/7 assay in the treated cultures after 24 h further supported the selective induction of apoptosis in 3D with double and triple inhibitor combinations and underscored the most profound cancer cell eradication by T/MK/AZD treatment (Figure 2F).

### 2.4. Cancer Cell Spheroid Outgrowth in Extracellular Matrix Confirms T/MK/AZD as the Most Potent Combination Treatment Option Analyzed

**Figure 3:**
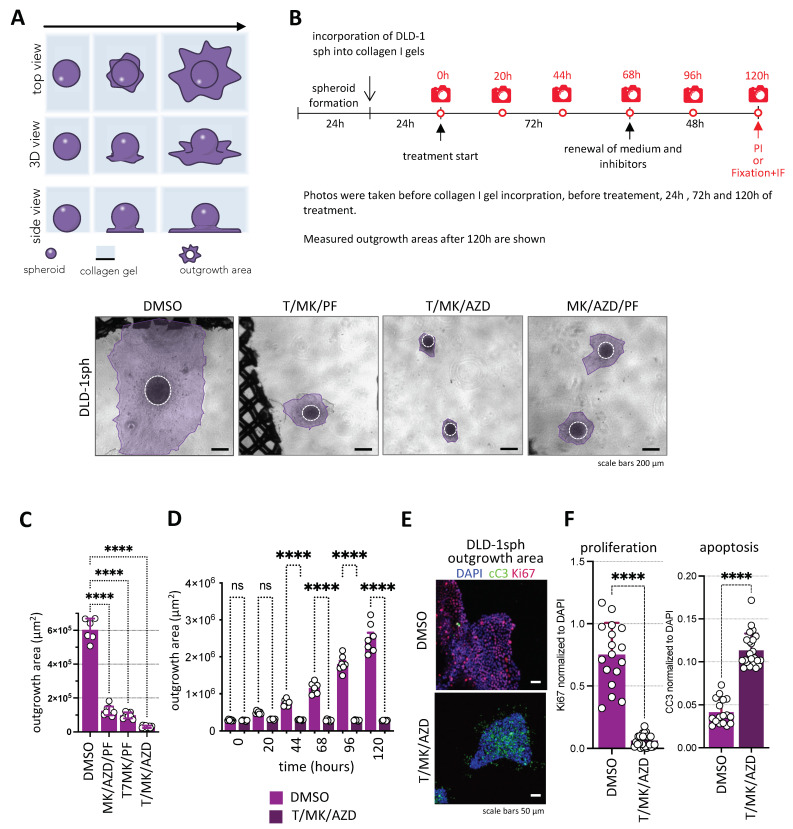
Colon cancer cell outgrowth on the surface of collagen I gels. (**A**) Schematic representation of the assay. (**B**) Overview of the time frame of the experiment. (**C**) DLD-1 spheroids were cultured in collagen I gels for 24 h and subsequently treated with triple-inhibitor combinations of Torin1 (250 nM), PF4708671 (10 µM), MK2206 (1 µM) and AZD6244 (1 µM) for 5 days. DMSO served as control. (**C**) Representative phase contrast microscopic images of spheroids (encircled) and cancer cell outgrowth areas (purple) on the surface of the collagen I gels are shown. Scale bars represent 100 µm. (**C**) After 72 h of treatment the outgrowth areas of DLD-1 spheroids on the collagen I gel was measured. Total outgrowth areas were normalized to the respective DMSO controls (*n* = 4–14 per condition). P-values are indicated (One-way ANOVA post normality test). (**D**) Spheroid outgrowth area (in µm^2^) over time in DMSO vs. T/MK/AZD. (**E**) Representative confocal immunofluorescence pictures showing Ki67 (red) and cleaved caspase 3 (cC3, green) levels in spheroid outgrowth regions under T/MK/AZD compared to DMSO controls. Cell nuclei in blue (DAPI). (**F**) Quantification of mean fluorescence intensities (MFI) of cC3 and Ki67 normalized to DAPI in five different spheroids at three different optical sections each in confocal images. Error bars are SD, dots indicate fluorescence intensity of individual images. *p* values are indicated by asterisks.

In order to simulate a biologically more relevant in vivo situation where cancer cells are embedded in extracellular matrix, we next cultured the CRC cell spheroids in collagen I gels. Using this robust 3D colon cancer model (Figure 3A), reminiscent of tumor cell migration in vivo [21], we analyzed the response of DLD-1 cells in collagen I gels under treatment with the selected inhibitor combinations. Spheroids were seeded into collagen gels and cultivated for 24 hrs. Subsequently, they were treated with the inhibitors and photographed after 1–5 days of culture (Figure 3B), and tumor cell outgrowth was quantified (Figure 3C). Again, T/MK/AZD treatment resulted in the most effective inhibition of spheroid outgrowth reaching less than 10% of the DMSO control, whereas MK/AZD/PF and T/MK/PF resulted in about 75% and 80% reduction of spheroid outgrowth area at 68 hrs of treatment. Thus, by the robust and most efficient eradication of in vitro tumor growth and spreading, we decided to further focus on T/MK/AZD treatment. Indeed, in an additional experiment, we could demonstrate the complete abolition of tumor cell outgrowth by T/MK/AZD over 5 days in comparison to the control (Figure 3D). The inhibitory effect was independent of the culture time of spheroids in collagen gels prior to treatment (Supplementary Materials Figure S3). Confocal immunofluorescence microscopy of cC3 in the DLD-1 spheres and outgrowth areas confirmed the ample increase in apoptosis under treatment previously seen in the spheroids. Moreover, the proliferation marker Ki67 profoundly decreased upon treatment, hardly showing one proliferative cell in the treated cultures (Figure 3E,F).

Thus, we concluded that triple-inhibitor treatment targeting mTOR, AKT and the MAPK pathway efficiently abolished cell proliferation and substantially eradicated tumor cells by apoptosis in different 3D cancer model systems.

### 2.5. A Dual Pan PI3K and mTOR Inhibitor, with Promising Clinical Potential Could Replace the Experimental Drugs Torin1 and MK2206

**Figure 4:**
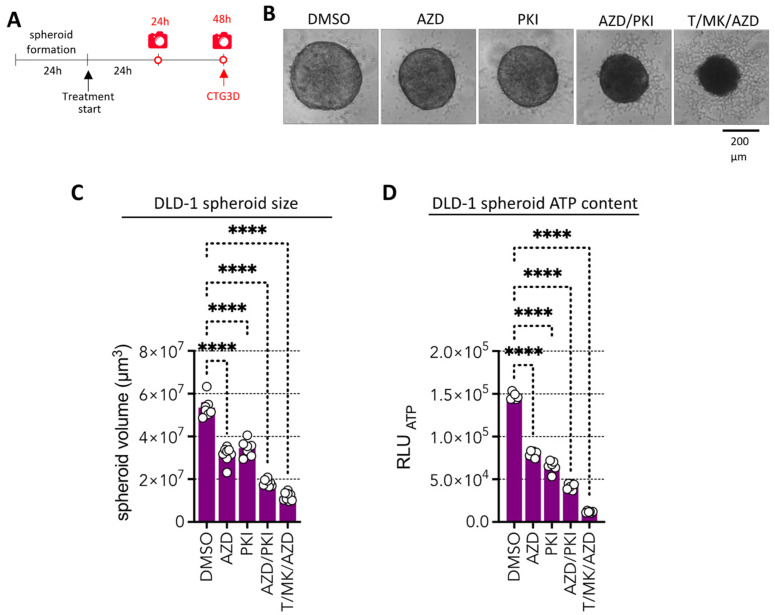
Impact of the pan PI3K/mTOR inhibitor gedatolisib (PKI-587, PF 05212384) alone or combined with AZD on DLD-1 spheroids in comparison to T/MK7AZD. (**A**) Overview of the time frame of the experiment. (**B**) Representative phase contrast images of DLD-1 spheroids treated for 48 h with the indicated inhibitors. (**C**) Spheroid volume after 48 h of treatment. (**D**) Cellular ATP levels determined with CellTiter-Glo 3D (CTG3D) after 48 h of spheroid treatment. Luminescence values (RLU_ATP_) were determined (*n* = 6 per condition). Bar graphs are mean + standard deviation (SD); individual values are shown (circles). *p* values are indicated by asterisks.

Next, we aimed to reduce the numbers of inhibitors and to select molecules which show promising clinical response and safety in clinical trials. The MEK inhibitor (AZD, AZD6244, selumetinib) used in this study is already FDA-approved for the treatment of type I neurofibromatosis in children. However, Torin 1 was never used in clinical trials and MK2206 was used in 50 clinical trials but did not reach approval so far. Thus, we selected PKI-587 (PKI, gedatolisib), a highly potent dual panPI3K/mTOR inhibitor [22] which was granted fast FDA track designation [23] and breakthrough therapy designation [24] in 2022 for HR^+^/HER2^−^ breast cancer that failed to respond to CDK4/6 therapy. We hypothesized that this high potential inhibitor, which inhibits AKT activity (since PI3K is upstream of AKT) and simultaneously mTORC 1 and 2 kinase activity, might be equally potent in our assay as compared to the combination of MK2206 and Torin1. In order to test this, we treated DLD-1 colon cancer spheroids with AZD and PKI individually and in combination and compared it also to the most efficient T/MK/AZD triple combination for 48 h (Figure 4A). Indeed, AZD/PKI treatment induced similar changes in spheroid appearance as T/MK/AZD reminiscent of dying cells (Figure 4B), whereas AZD- or PKI-treated spheroids morphologically appeared similar to the control, however, were smaller. Quantitative assessment of spheroid volume corroborated this observation (Figure 4C), revealing significant differences. Assessment of ATP content, as a measure of cellular health, revealed strong reduction of viability in the AZD/PKI combi treatment, indicating effective cell killing (Figure 4D). However, T/MK/AZD treatment was significantly more effective than the PKI/AZD combination. Hence, we decided to further evaluate these drugs in a different and clinically more relevant setting.

### 2.6. Combined PKI-587 and AZD Treatment of Patient-Derived Colon Cancer Organoids Is Comparably Efficient as T/MK/AZD Treatment

Organoids derived directly from patient tumors are far more closely recapitulating individual cancer morphology, drug response and personalized patient tumor characteristics than tumor cell lines, even if they are grown in 3D as multicellular spheroids. Most importantly, the mutational landscape of the individual patient tumors is maintained in the organoids. Taken together, these features make organoids the ideal tools to study personalized drug responses in vitro.

Thus, this physiologically relevant system was used for further evaluation of the drug combinations selected. For this, organoids from three different patients were isolated and expanded using standard culture techniques [25]. Subsequently, these organoids were harvested and seeded on top of Matrigel coated flat collagen gel cylinders and cultivated in cell culture inserts at the air liquid interface (Supplementary Materials Figure S4). This setup guarantees that all organoids adhere at the top of the Matrigel-covered collagen gel, ensuring microscopic imaging in one optical plane. Hence, the growth of organoids can be monitored over time by simple phase contrast microscopy with all organoids being in focus. This allows temporal organoid growth in selected areas to be followed (microscopic frames at the same position over time).

**Figure 5:**
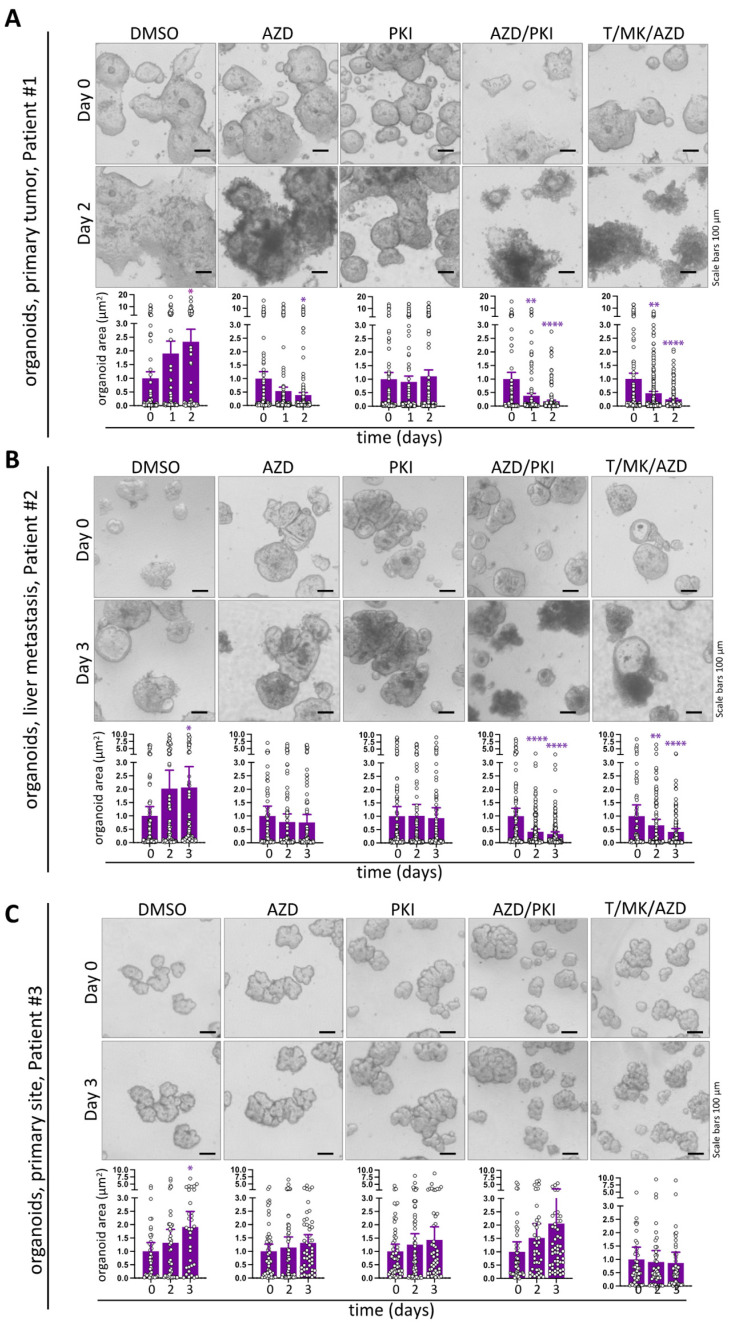
Impact of single and combined PKI-587 and AZD treatment on organoids in comparison to T/MK/AZD treatment. (**A**) Representative phase contrast images of a primary human organoids for 48 h with (combi)inhibitors. The size of every individual organoid present in three microscopic frames was determined over time and plotted (dots). Bars represent mean organoid projected area over time. Error bars show SEM. (**B**) Images and mean organoid size of human colon cancer organoids derived from liver metastasis treated and analyzed as in (**A**). (**C**) Images and mean organoid size of colon cancer organoids from a primary colon cancer of a different patient treated and analyzed as in (**A**). Significant changes to the corresponding d0 time points are indicated. *p-*values are shown (asterisks, purple). Non-significant changes were not labeled.

For the drug response studies, organoids from the three different patient tumors were harvested, seeded and incubated for four days without treatment to recover from trypsinization. Thereafter, the cultures were treated for 48 h with AZD and PKI individually and in combination as well as with T/MK/AZD; DMSO served as solvent control. Treatment induced profound organoid disintegration after 48 h as evident in phase contrast microscopy images from two patients (Figure 5A, colon cancer from primary site; Figure 5B, colon cancer liver metastasis). The changes in phenotypic appearance, i.e., living, bright organoid areas in contrast to dark-appearing, dead areas, were quantitatively confirmed by mean organoid size (living organoid projected area) measurements over time (Figure 5A,B). Organoid size was profoundly reduced under PKI/AZD and T/MK/AZD combi treatment, showing equal efficiency in size reduction, whereas AZD and PKI single treatment was less effective. Importantly, organoids grew in size when treated with DMSO solvent control.

Interestingly, in organoids from a patient with primary colon cancer, no collapse of organoid structures was evident (Figure 5C) and organoid morphology was indistinguishable from DMSO controls. This was accompanied by a lack of size decrease (T/MK/AZD) or even increase in organoid size (AZD, PKI, AZD/PKI) over time (Figure 5C).

### 2.7. Response to Treatment Is Associated with Increased Apoptosis and Decreased Proliferation in Patient Derived Colon Cancer Organoids

**Figure 6:**
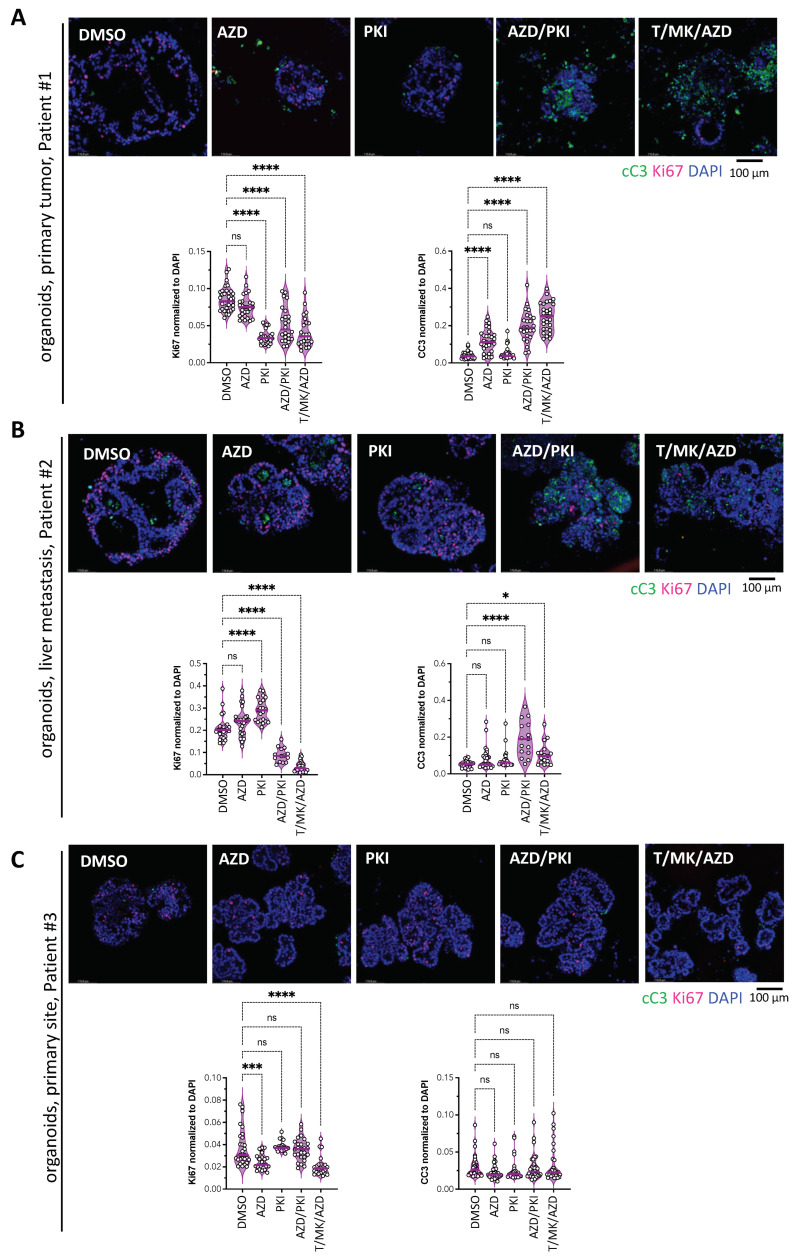
Analysis of proliferation and apoptosis in colon cancer organoids under treatment. Representative confocal immunofluorescent images of the human organoids from Figure 5 after 48 h of treatment stained for Ki67 and cC3 to assess proliferation and apoptosis. Quantification of mean fluorescence intensities (MFI, bars) of cC3 and Ki67 normalized to DAPI in five different organoids at three different optical sections each. Dots in violin plots indicate fluorescence intensity of individual images. (**A**) Organoids from the primary colon cancer from patient #1, (**B**) liver metastatic colon cancer organoids form patient #2. (**C**) Primary colon cancer organoids from patient #3. Asterisks indicate significant changes; ns, non-significant.

Next, proliferation and cell death were analyzed in the organoid cultures from Figure 5. They were fixed after two days of treatment and subjected to whole-mount immunofluorescence staining with the apoptosis marker cC3 and the proliferation marker Ki67 and subsequent confocal imaging (Figure 6A–C). Quantitative image analysis revealed that in organoids from patient #1, PKI, PKI/AZD and T/MK/AZD significantly reduced proliferation, whereas AZD, PKI/AZD and T/MK/AZD increased cell death. In the metastasis-derived organoids from patient #2, a significant decrease under AZD/PKI and T/MK/AZD was accompanied by elevated apoptosis. In organoids derived from the primary colon cancer of patient #3, which did not show any organoid dissolution in phase contrast images (compare to Figure 5C), indeed, apoptosis remained low in all conditions without significant changes upon treatment (Figure 6C). Ki67 signals only showed subtle changes with reaching significance under AZD and T/MK/AZD conditions.

### 2.8. Mutations in the PI3K and/or RAS-RAF-MAPK Pathway Account for Responsiveness to Treatment

**Figure 7:**
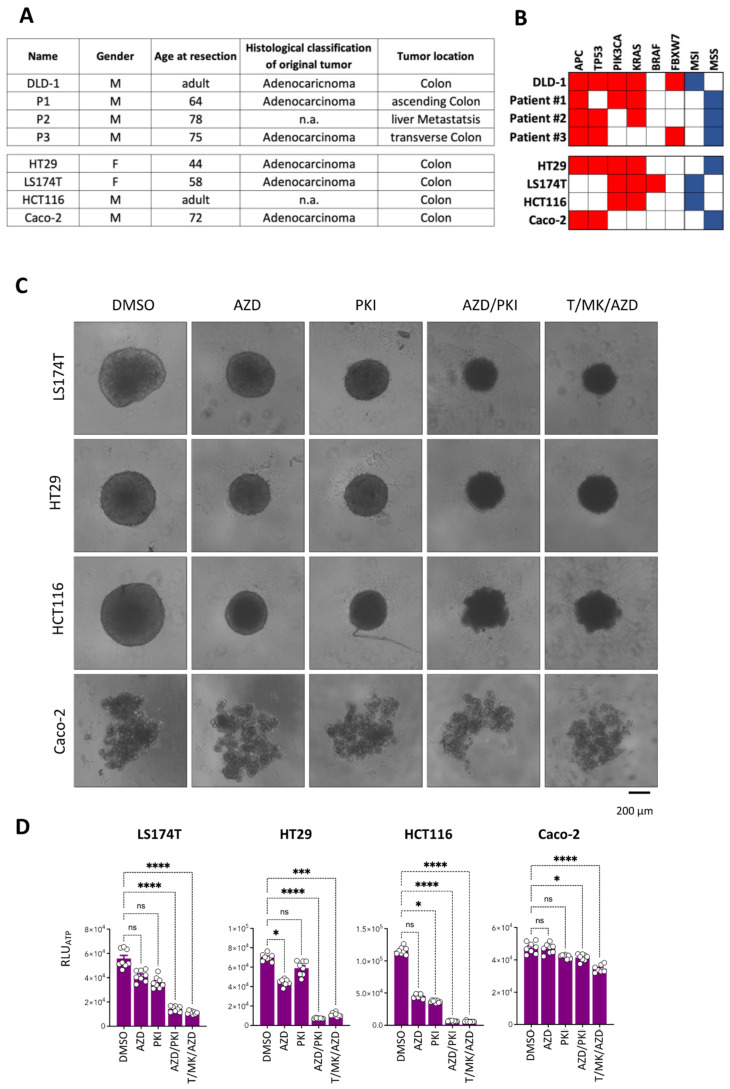
Treatment response correlates with mutational status in PI3K and/or KRAS. (**A**) Cell line and patient information. (**B**) Mutational (red) and microsatellite status (blue) of DLD-1 cells and the tumors from three patients (#1, #2, #3) of the most commonly mutated genes in CRC of which the organoids were derived (top panel). Mutations in other widely used CRC cell lines (bottom panel) used in this study. (**C**) Representative phase contrast microscopic images of spheroids of the indicated tumor cell lines treated with single inhibitors and combinations for 48 h. DMSO cultures are controls. Scale bar is 200 µm. (**D**) Cellular ATP levels determined with CellTiter-Glo 3D (CTG3D) after 48 h of spheroid treatment. Luminescence values (RLU_ATP_) were determined (*n* = 8 per condition). Bar graphs are mean + standard deviation (SD), individual values are shown (circles). *p*-values are indicated by asterisks.

To understand the reason for why patient #3 was resistant to treatment, we compared the mutational profile of the most commonly altered genes in CRC in the DLD-1 cancer cells with the mutational landscape of the three tumors from the patients. Strikingly, patient #3 displayed no mutations in PIK3A, KRAS or BRAF, whereas patient #1 and the DLD-1 cells had mutations in both PIK3A and KRAS, and the liver metastasis of patient #3 showed a KRAS mutation (Figure 7A,B). Hence, we hypothesized that we could predict effective treatment response by the presence of a mutation in either the PI3K/MTOR pathway or the RAS-RAF-MAPK pathway. A set of commonly used colon cancer cell lines was selected on the basis of their published mutational profiles. CaCo-2 cells were devoid of mutations in PIK3A and KRAS or BRAF, whereas HT29, HCT116 and LS174T displayed a combination of PI3KA and KRAS mutations. Based on our hypothesis, Caco-2 cells should be non-vulnerable to the treatment conditions, whereas all other cell lines should be.

Indeed, after two days of treatment of HCT116, HT29 and LS174T spheroids, reduced spheroid size and morphological changes were detectable (Figure 7C), whereas CaCo-2, which form acinar cell structures in 3D, did not show any signs of reduced growth or phenotypic changes. Notably, viability (cellular ATP content) was profoundly reduced under PKI/AZD and T/MK/AZD treatment compared to the solvent control and single drugs in LS174T, HT29 and HCT116 cells. No difference was detected in response to the triple treatment or the optimized double treatment. Strikingly, Caco-2 spheroids remained viable and showed only subtle reduction in ATP content upon the two combination treatments (Figure 7D).

These results indicate that profound response to our novel efficient combination treatments is dependent on the presence of either PIK3A or KRAS mutations in the colon cancer specimens.

## 3. Discussion

We used 3D spheroid models and organoid cultures to assess the effectiveness of selected small molecule inhibitors and compared it to 2D models. These models are employed to be very close to the in vivo situation, but never can recapitulate the in vivo complexity of a living organism. However, in the meantime there is ample evidence that 3D cultures better mimic solid human tumors than 2D cultures [8,9,10]. Moreover, the biological relevance of these models for drug testing is well established [15,16,17] and further complemented by the precision medicine approach of patient-derived cancer organoids [18,19].

In this study, we found profound tumor cell destruction in CRC cell lines and organoids treated with a combination of T (Torin 1), MK (MK2206) and AZD (selumetinib) or by administration of PKI (gedatolisib) in concert with AZD. Both inhibitor combinations simultaneously target the PI3K/AKT/MTOR and the MAPK pathways. Treatment resulted in a profound reduction of cell proliferation both in 2D and spheroids, however, unexpectedly, cell death was induced only in 3D. Interestingly, MK/AZD/PF showed the best inhibitory effect in the ATP assay. This might be due to a pronounced effect on the metabolism/energy status of the cells. However, the morphology of the spheroids clearly showed that MK/AZD/PF had not the most prominent cell killing effect as recognizable by a smoother spheroid surface and a lighter spheroid area as compared to T/MK/AZD. This was further reflected by the lower PI, cleaved caspase 3 and ApoONE positivity (see Figure 2). Thus, we conclude that assessment of just one parameter is not enough to select for the most efficient anti-tumor drug combination and relied on the assessment of six different parameters: 1. ATP content, 2. spheroid size, 3. spheroid appearance, 4. PI positivity, cleaved caspase 3/PARP Western/caspase3/7 assay, 5. cC3/Ki67 IF, 6. Spheroid outgrowth in a 3D matrix to identify the most efficient inhibitor combination to destruct tumor cells.

In summary, signal transduction analysis by Western blotting revealed proper drug action under both conditions. Induction of cell death was due to apoptosis in the 3D colon cancer models. Moreover, treatment of primary patient-derived colon cancer organoids was similarly efficient and led to increased apoptosis and decreased proliferation in both combined PKI/AZD as well as T/MK/AZD treatment. To the best of our knowledge, this is the first report that spheroids are more prone to anti-cancer treatment than their 2D counterparts cultivated on plastic surfaces. This is unexpected, as it is well established that spheroids are less sensitive to chemo- or radiotherapy, a phenomenon called multicellular resistance, which is composed of contact resistance and resistance inherent to the 3D structure (e.g., less proliferation, quiescent cells, decreased drug penetration) of spheroids [13]. One potential reason for the decreased toxicity of chemo- and radio and targeted therapy in spheroids is related to apoptosis. In a study upon targeted inhibition with the proteasome inhibitor bortezomib, NOXA, a pro-apoptotic sensitizer was not upregulated in spheroids in contrast to 2D [26]. This could be reverted by the HDAC inhibitor vorinostat [27]. Whether, contrary to the above situation, upregulation of NOXA is active in our 3D setting under PI3K/MTOR/MEK inhibition and not in 2D remains to be analyzed in the future.

The identification of the cytostatic and cytotoxic effect of T/MK/AZD (selumetinib) or PKI (gedatolisib)/AZD in 3D in contrast to the sole cytostatic effect in 2D allows to draw two conclusions. First, the strong and rapid tumor cell killing might efficiently eliminate tumor cells in a short time frame, not allowing these cells to adapt and develop resistance mechanisms [3]. This is possibly an advantage over the majority of chemotherapies used in CRC, which need several cell divisions (i.e., incorporation of 5-FU into the DNA, or topoisomerase inhibition of irinotecan) to fulfill their cell-killing capacity. Second, our finding underscores the advantage of (combinatorial) screening with chemical libraries in 3D settings, an endeavor now more often undertaken by the pharmaceutical industry [28].

Mutational analysis of the patient organoids and the established cell lines revealed that the presence of PI3K and/or RAS-RAF-MAPK pathway mutations account for responsiveness to treatment. This is in concordance with earlier studies and current patient stratification strategies for cancer patient treatment both in clinical routine as well as in trials [29]. Interestingly, organoids from patient #1 are very well-responsive to MEK inhibition (AZD) alone. These organoids have mutations in both PIK3CA AND KRAS, whereas the organoids from patient#2 harbor mutations in TP53 and KRAS. Interestingly, the KRAS mutations are the same in both patients (Ala146Thr). Thus, there is still need to further characterize these organoids in more depth and add more patient samples. This is an aim we will address in the future. The non-responsiveness of cancer cell lines and organoids with other mutations but lacking PI3K and MAPK pathway alterations might suggest that the treatment combination will also not affect normal (isolated from healthy mucosa) organoids lacking any mutations, thereby leading to the assumption of low damage to the healthy tissue of the targeted organ. This hypothesis clearly needs further validation by in vitro studies using normal intestinal organoids in the future and in vivo studies in mice.

Caco-2 cells, which were resistant to the PKI/AZD and T/MK/AZD treatments are widely used in gastrointestinal toxicity tests and absorption studies and a well-accepted in vitro model of absorptive enterocytes [30]. Despite the fact that these cells are cancer cells, when seeded on filter inserts, they can be differentiated to a monolayer of polarized cells with tight junctions. Interestingly, Caco-2 spheroids displayed small multiple spheroid aggregates in our spheroid assay, which is not reported in some other studies [31,32]. A careful review of the suggested papers revealed that our spheroid formation protocol differs from the ones described in these two papers. We form our spheroids in low-attachment 96 well plates with 0.3% methylcellulose in DMEM 10% FCS, whereas Gheytanchi et al. used hanging drops in serum-free medium and Varesano et al. used ultra-low attachment plates. However, both studies used serum-free DMEM/F12 supplemented with EGF. This might explain the difference in morphology of the spheroids in our setup compared to the others.

Four MEK inhibitors are meanwhile FDA approved to treat melanoma, neurofibroma, NSCLC and thyroid cancer in stratified patients with BRAF or KRAS mutations. Unfortunately, there is an inevitable development of acquired resistance against MEK inhibitors [33]. Some PI3K inhibitors have also been approved for certain types of leukemia, lymphoma and breast cancer. However, these treatments are often associated with severe side effects and in the case of PI3K inhibitors raised strong safety concerns in the FDA [34,35]. Nevertheless, several preclinical studies and clinical trials show promising anti-tumor activities in simultaneously targeting PI3K and MEK [36,37,38,39]. Thus, with the current treatment options, increased efficacy of PI3K/MAPK pathway combinatorial inhibition is achieved at the expense of augmented toxicity [40].

Targeted drug delivery strategies are a promising route to avoid severe side effects and to efficiently localize the compounds to the tumor site [41]. Advances in drug delivery approaches led to improved pharmacological parameters, without compromising selective drug action on the molecular target. In this way pharmacokinetics, stability, biodistribution, absorption of the drug and its exposure to tumors and healthy tissues can be modulated [42], thereby also improving synergistic combination treatments. Thus, this field of biomedicine offers a personalized approach and simultaneously avoids undesired toxicities, which would improve quality of life of the treated patients. Combined with the approach of combined inhibition of major cellular pathways, the targeted delivery strategy promises great potential to efficiently and selectively eradicate cancer cells. In this study, we identified the combination of selumetinib and gedatolisib to provide strong anti-cancer activity in a preclinical setting using primary 3D cultures. Since one of these molecules has been FDA approved, whereas the other one is on fast track for FDA approval and their toxicology and efficacy profiles in humans are established, our results might initiate clinical trials to assess these drugs in colon cancer patients in the near future.

## 4. Material and Methods

### 4.1. Cells and Cell Culture

Human colon cancer cell lines were obtained from American Type Culture Collection (ATCC, Manassas, VA, USA). DLD-1 (CCL-221™), LS174T [CL-188™)], HT29 (HTB-38™), HCT116 (CCL-247™) and Caco-2 (HTB-37™) were expanded and used at early passages. Cells were authenticated by short tandem repeat (STR) profiling. The cells were cultivated in Dulbecco’s modified Eagle’s medium (DMEM), high glucose (4.5 g/l) supplemented with 10% fetal calf serum, 2 mM L-glutamine (Gln) and antibiotics [60 mg/L penicillin, 100 mg/L streptomycin sulfate (PS)] at 37 °C in 5% CO2. Spheroid formation (3D culture) with 3000 cells per spheroid was induced as described [14]. In brief, cells were detached, counted and seeded in 100 μL of sterile filtered DMEM with 5% FCS/glutamine/PS containing 0.3% methylcellulose into 96-well plates (round bottomed, untreated) for 24 h (DLD-1) or 48 h (HT-29, LS174T, HCT116). For 2D analysis in 96 well plates 3000 (mono- and double-inhibitor combinations) or 6000 cells (triple inhibitors) per well were used. Cells were cultured at 80% humidity, under 5% CO2 and normoxia.

### 4.2. Spheroid Outgrowth Assay

Spheroid outgrowth was performed in collagen I gels as described [8]. For one gel 48 spheroids were harvested, resuspended in 300 µL neutralized collagen 1 solution and poured into silicone casting molds and a polyester mesh (mesh size: 100 µm, fiber diameter: 77 µm, 20000194, Bückmann GmbH and Co, Mönchengladbach, Germany) was submerged and incubated 5 min on ice to let the spheroids sink to the bottom by gravity. Thereafter, polymerization of collagen I gels was performed at 37 °C for 20 min. The collagen I gels were carefully transferred into 12-well tissue culture plates (CC7682-7524, CytoOne^®^, Starlab, Hamburg, Germany) already containing 2 mL of cell culture medium and incubated as free-floating gels and treatments started. Spheroids and outgrowth areas, emerging from the spheroids at the lower collagen gel surface were photographed daily and quantified in ImageJ (for a schematic overview see Figure 3A).

### 4.3. Organoid Cultures

Organoids were expanded as described [43], harvested and cultured on Matrigel-coated collagen gels at the air liquid interface. Collagen gels from rat tail I (Corning, Corning, NY, USA #354236) were poured into silicon forms at concentration 2 mg/mL. A nylon mesh was inserted into the gel for stability during handling. When gels had solidified (after 20 to 30 min at 37 °C), they were coated with BMA by placing them on a 10 µL drop of Matrigel in a cell culture dish for 15–20 min and then lifted on cell culture inserts (Millicell, 0.4 µm, 30mm diameter; Merck Milipore Ltd.,Burlington, MA, USA), with the Matrigel coating facing upwards. Organoids were plated on top of the collagen gels at 50 organoids/gel. The cultures were positioned in 60 mm dishes and 1.5 mL medium was added to each well so that the organoids were grown at the air-liquid interphase. The cultures were allowed to expand for 1 week, after which they were washed with ice cold PBS and fixed in 4% paraformaldehyde for 20 min. The cultures were stained with immunofluorescence for confocal microscopy. Patient material for organoid isolation was collected with informed consent and collection was approved by the ethics review board of the Medical University of Vienna (N# 1248/2015).

### 4.4. Inhibitor Treatment

Cultures were treated with different inhibitors. Cellular and spheroid morphology was evaluated microscopically (Olympus, Tokyo, Japan) and was analyzed in ImageJ to determine spheroid volume by measuring projected areas, followed by radius and volume (μm³) determination or spheroid outgrowth. For inhibitor treatment in 2D and 3D cultures, 250 nM of Torin1 [44], 10 μM of PF47068671 [45], 1 μM of MK2206 [46] and 1 μM of AZD6244 [47] were employed as these concentrations have been most frequently used in cell-based assays and have a complete effect on their respective targets (phospho-protein analysis in Western blots, Figure 1F). For inhibitor details see Table 1.

### 4.5. Metabolic Activity and Caspase 3/7 Activity Assay

The metabolic capacity of cells was determined by measuring cellular ATP levels with a luciferase based CellTiterGlo 3D (Promega) according to the manufacturer’s protocol and luminescence measurement (Synergy HT, Biotek, Winooski, VT, USA^)^. Caspase3/7 activity in the cells was determined using the Apo-ONE Homogeneous Caspase-3/7 Assay (Promega) using an a profluorescent consensus substrate, rhodamine 110 bis-(N-CBZ-l-aspartyl-l-glutamyl-l-valyl-aspartic acid amide; Z-DEVD-R110) according to the instructions from the manufacturer.

### 4.6. Western Blot Analysis

Cell lysates were prepared from conventional cultures or spheroids. Cells and spheroids were washed in cold PBS and lysed in RIPA lysis buffer (50 mM Tris-HCl pH 7.6, 150 mM NaCl, 1% TritonX-100, 0.1% SDS, 0.5% sodium deoxycholate, 1 mM PMSF, 4 μg/mL aprotinin, 4 μg/mL leupeptin, 0.6 μg/mL benzamidinchloride, 20 μg/mL trypsin inhibitor). Supernatants were collected after centrifugation (20 min, 18,800 g, 4 °C). Equal amounts of protein (15 μg) were denatured in loading dye (200 mM Tris-HCl pH 6.8, 400 mM DTT, 8% SDS, 0.4% bromophenol blue, 40% glycerol), subjected to SDS-PAGE and transferred to nitrocellulose membranes. Blocked membranes were probed with primary antibodies at 4 °C overnight and with horseradish-peroxidase (HRP)-conjugated secondary antibodies (anti-mouse-IgG heavy and light chain, and anti-rabbit-IgG heavy and light chain, Bethyl Laboratories, Montgomery, TX, USA) at RT for 1 h. Signals were detected using chemiluminescence and X-ray films. The mean integrated density of each band was determined with ImageJ (National Institutes of Health). For antibodies, see Table 2 and Table 3. The assessment was done in a single experiment to document proper drug action as in [14], but was not intended as a general statement of fold changes upon different conditions.

### 4.7. Immunofluorescence

Cells, spheroids or collagen gels were fixed in 4% paraformaldehyde (PFA) at room temperature for 30 min. For sectioning, fixed spheroids were molded into agarose gels (1%), embedded into paraffin, sectioned (5 μm) and subjected for antigen retrieval in DAKO citrate buffer pH 6 (Agilent, Santa Clara, CA, USA) at 120 °C for 10 min. In 2D culture, cells fixed in PFA (4%) were permeabilized in methanol at −20 °C for 10 min. 2D and 3D culture samples were blocked in PBS with 1% BSA at room temperature for 1 h. Primary antibody incubation was performed at 4 °C overnight. Alexa-Fluor^®^488- and Alexa-Fluor^®^647-conjugated secondary antibodies (Thermo Scientific, Waltham, MA, USA) were used at room temperature for 1 h. Nuclei were counterstained with 2 μg/mL DAPI (Sigma). Slides were mounted with Vectashield^®^ (Vector Laboratories, Burlingame, CA, USA). For antibodies used, see Table 1 and Table 2. Confocal fluorescence images were recorded on a Leica-SP8 with a 20x objective (NA = 1.3). For collagen gel immunofluorescence in situ, the fixed gels were permeabilized in 80% ethanol for 1 h, washed two times in TBS/0.1% Triton X100, and three times in PBS/0.1%Triton for 10 min each at RT. Gels were blocked in PBS/1% BSA for 3 h at RT, and incubated with primary antibodies in PBS/BSA for 24 h at 4 °C. Gels were washed 3x in PBS/T for 15 min, secondary antibody was added in PBS/BSA for 24 h and washing repeated. DAPI (2 μg/mL) was added for 3 h in PBST, washed and gels were imaged on the Leica confocal microscope at different optical planes.

### 4.8. Image Analysis

For Ki67 and cleaved Caspase 3 (CC3) quantification after confocal imaging, the raw integrated density (RID) of each optical section, frame and channel (red: Ki67; green: CC3; blue, DAPI) was determined in ImageJ. The background of each channel was determined by imaging empty gels or slides and subtracted. Ki67 and CC6 RID was divided by the DAPI RID to normalize for total cell numbers present in the pictures. A minimum of four regions of the sample were analyzed in a minimum of four optical sections with a distance of 20 µm each. For organoid growth over time, phase contrast images of randomly selected regions were taken and these regions were followed over time for 3–4 days. The size of every individual living organoid structure (omitting dead, dark-appearing regions) present in three microscopic frames were determined in ImageJ using the freehand tool and a drawing tablet (Wacom, Intuos Pro) over time and plotted.

### 4.9. Statistical Analysis

Graphs were generated and statistical analysis was performed using GraphPad Prism 9. Bar graphs are presented as mean ± s.d. or s.e.m. For statistical analysis, Kolmogorov–Smirnov normality tests were performed followed by ordinary one-way ANOVA. *p*-values are indicated as * *p* ≤ 0.05, ** *p* ≤ 0.01, *** *p* ≤ 0.001 or **** *p* ≤ 0.0001.

## Figures and Tables

**Table 1:** Small molecule inhibitors used in the study. All inhibitory compounds are listed concerning their target of inhibition, the final concentration used in the study. Additionally, the product number and the company are given.

Name (Abbreviation)	Alternative Name	Target of Inhibition	Concentration Used	Product #	Company
**Torin1 (T)**		MTOR	0.25 µM	4247	Tocris Bioscience, Bristol, UK
**PF4708671 (PF)**		S6K1	10 µM	4032	Tocris Bioscience, Bristol, UK
**MK2206 (MK)**		AKT1/2/3	1 µM	S1078	Selleckchem, Munich, Germany
**Selumetinib (AZD)**	AZD6244	MEK1/2	1 µM	S1008	Selleckchem, Munich, Germany
**Gedatolisib (PKI)**	PKI-587	PIK3A/MTOR	0.5 µM	S2628	Selleckchem, Munich, Germany

**Table 2:** Secondary antibodies used in Western blot analysis and Immunofluorescence. Secondary antibodies used in this thesis are listed concerning their name, source, dilution ratio.

	Source	Dilution	Product Number
**anti-rabbit IgG-heavy and light chain antibody, HRP conjugate**	goat	1:10,000	A120-101P
**anti-mouse IgG-heavy and light chain antibody, HRP conjugate**	goat	1:10,000	A90-116P
**Alexa Fluor 488**	goat	1:500	A11008
**Alexa Fluor 647**	donkey	1:500	A31571

**Table 3:** Primary antibodies used in Western blot and immunofluorescence analysis. A list of primary antibodies used in this study depicting the name of the antibody, source, dilution ratio, product number and source. Cell signaling technology (CST; Danvers, MA, USA); Abcam (Cambridge, UK); SigmaAldrich (St. Louis, MO, USA); R&D Systems (Minneapolis, MN, USA).

	Clone	Source	Dilution	Product Number	Company
**p-Akt T308-XP^®^**	D25E6	rabbit	1:1000	#13038	CST
**p-Akt S473-XP^®^**	D9E	rabbit	1:1000	#4060	CST
**Akt pan**	40D4	mouse	1:2000	#2920	CST
**p-PRAS40 T246**	-	rabbit	1:1000	#2640	CST
**4E-BP1**	-	rabbit	1:1000	#9452	CST
**p-PKC (pan) β S660**	-	rabbit	1:1000	#9371	CST
**p-ERK1/2 T202/Y204-XP^®^**	D13.14.4E	rabbit	1:1000	#4370	CST
**ERK1/2**	137F5	rabbit	1:1000	#4695	CST
**p-p70 S6K T389**	108D2	rabbit	1:1000	#9234	CST
**p70 S6K c-term**	-	rabbit	1:1000	#9202	CST
**p-rpS6 S240/244**	-	rabbit	1:1000	#2215	CST
**rpS6**	54D2	mouse	1:1000	#2317	CST
**p-Rb S807/811**	-	rabbit	1:1000	#9308	CST
**IRS1**	-	rabbit	1:1000	#2382	CST
**E-Cadherin**	HECD-1	mouse	1:1000	ab1416	Abcam
**α Tubulin**	DM1A	mouse	1:5000	CP06	SigmaAldrich
**GAPDH**	-	rabbit	1:10,000	2275-PC-100	R&D Systems
**Ki67**	8D5	mouse	1:1000	#9449	CST
**Cleaved Caspase 3 (CC3)**	5A1E	rabbit	1:1000	‘9664	CST

## Data Availability

All data for this study are shown in the main Figures and supplemental Figures. There are now further data available.

## Supplementary Materials

The following supporting information can be downloaded at: https://www.mdpi.com/article/10.3390/ijms24021668/s1.
