# Supplementary material for: Selective Eradication of Colon Cancer Cells Harboring PI3K and/or MAPK Pathway Mutations in 3D Culture by Combined PI3K/AKT/mTOR Pathway and MEK Inhibition
Source: Int J Mol Sci. 2023 Jan 14;24(2):1668. doi: 10.3390/ijms24021668 (PMC9863259; doi:10.3390/ijms24021668)
Supplement: Supplementary file 1 [file ijms-24-01668-s001.zip › ijms-2059576-supplementary.pdf]

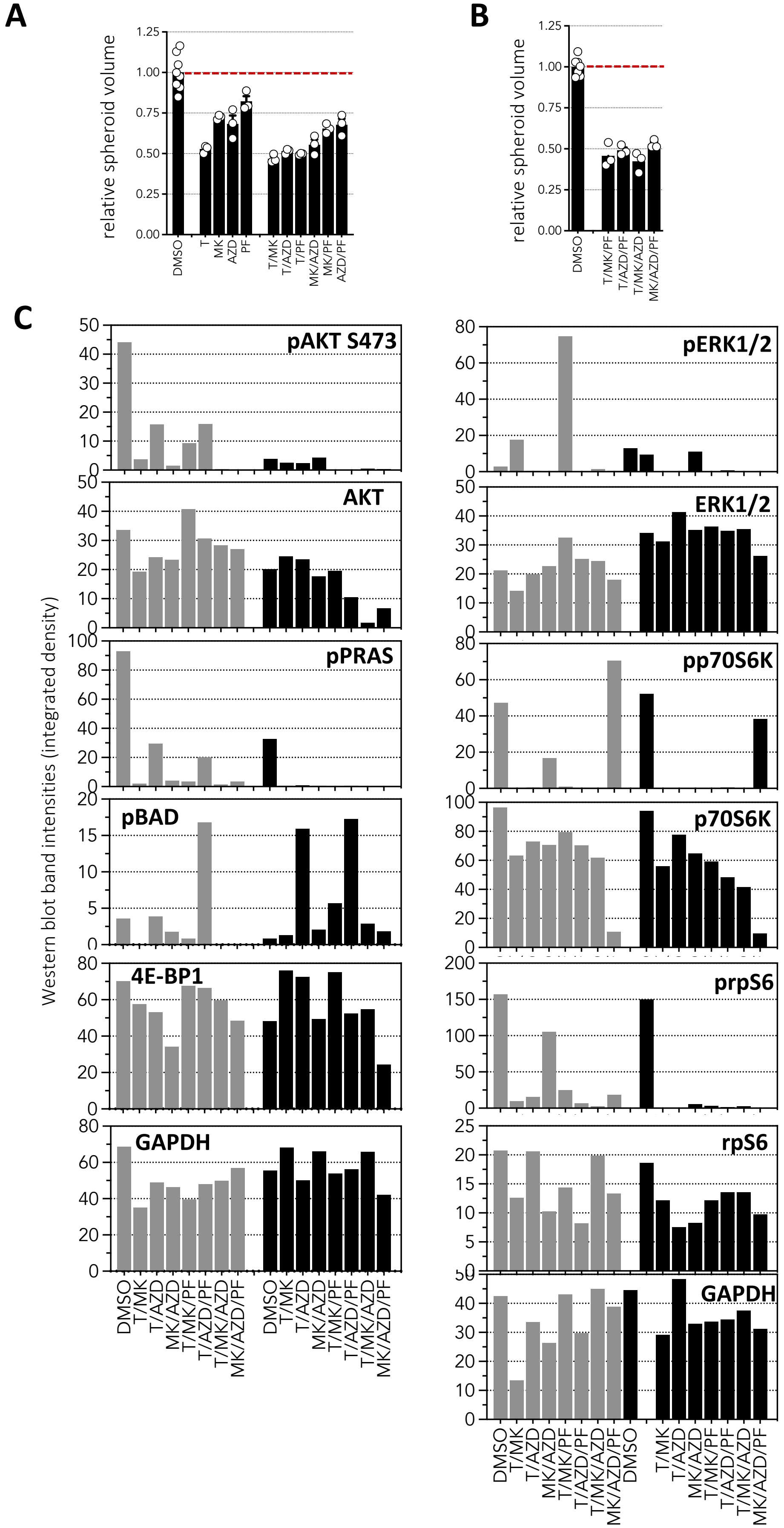

Supplementary Figure S1. *Spheroid volume and Western blot quantification.* **A** DLD-1 spheroids from Figure 1B,C were analyzed and projected areas were measured in ImageJ and the radius and volume was calculated. The mean spheroid volume is plotted in relation to the DMSO control (n=8 for DMSO, n=3 for treatments). **B** DLD-1 spheroids volumes from Figure 1D,E were determined as in A. The mean spheroid volume is plotted in relation to the DMSO control (n=7 for DMSO, n=3 for treatments). **C** Western blot densitometric quantification from blots shown in Figure 1F. Integrated densities of background adjusted bands are shown.

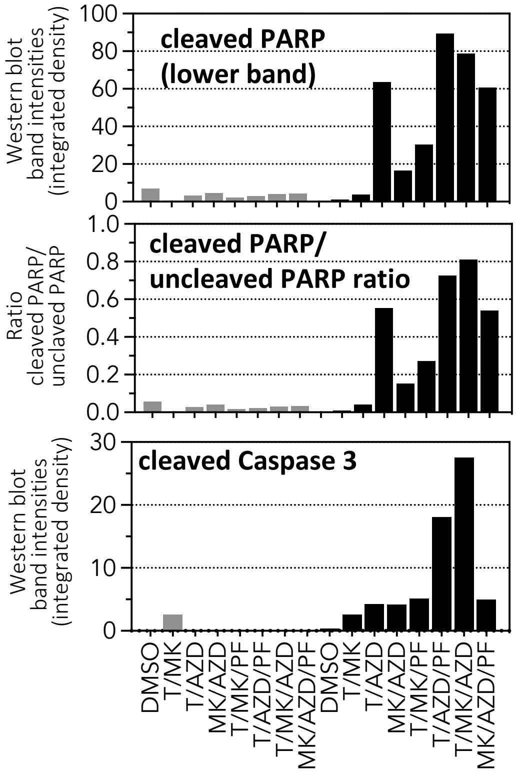

Supplementary Figure S2. *Western blot quantification.* **C** Western blot densitometric quantification from blots shown in Figure 2E. Integrated density of background adjusted bands are shown. In case of PARP the ratio of claved PARP vs uncleaved PARP is also shown.

A

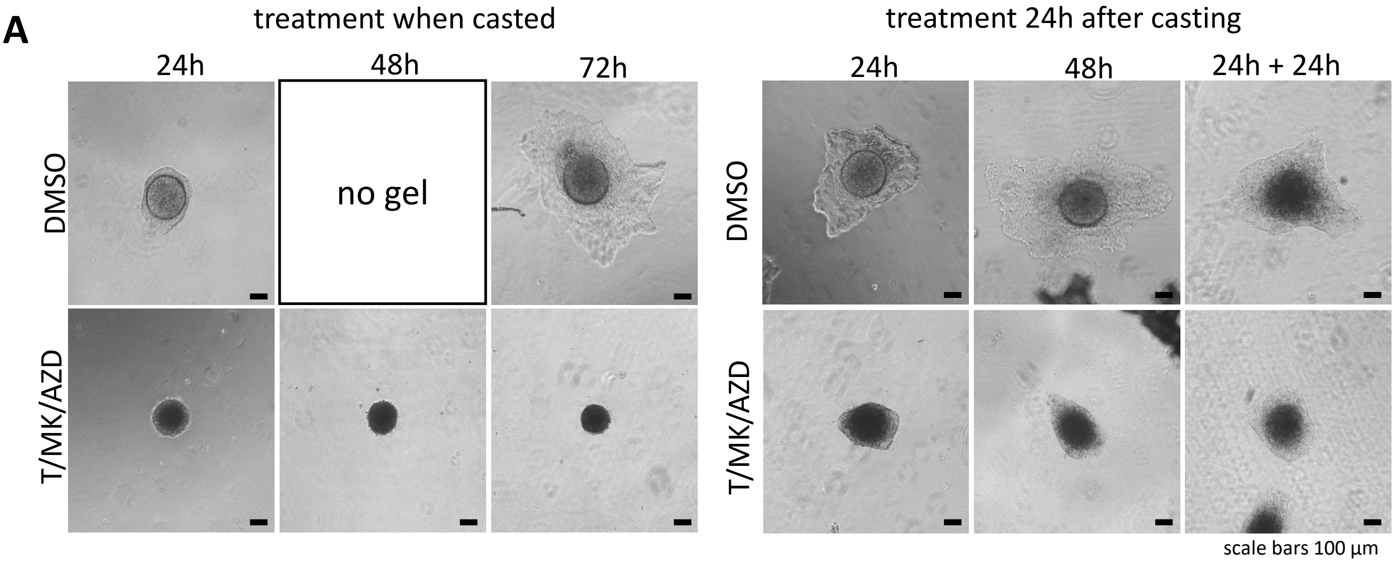

B

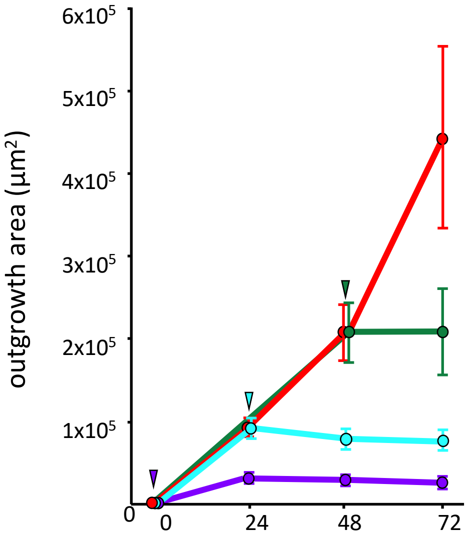

Supplementary Figure S3: Outgrowth of DLD-1 spheroids in collagen gels. DLD-1 spheroids were cultured in collagen I gels and immediately or subsequently treated with triple inhibitor combinations of Torin1 (250 nM), MK2206 (1 µM) and AZD6244 (1 µM) for 3 days. DMSO served as control. **A** Representative phase contrast microscopic images of spheroids and outgrowth areas on the surface of the collagen I gels are shown. Scale bars represent 100 µm. **B** **T**he outgrowth areas of DLD-1 spheroids on the collagen I gel were measured over time. Total outgrowth areas were normalized to the respective DMSO controls (n = 4-14 per condition). Arrowheads indicate treatment starts, purple: 0 h; cyan, 24 h; Green 48 h; red, untreated DMSO control.

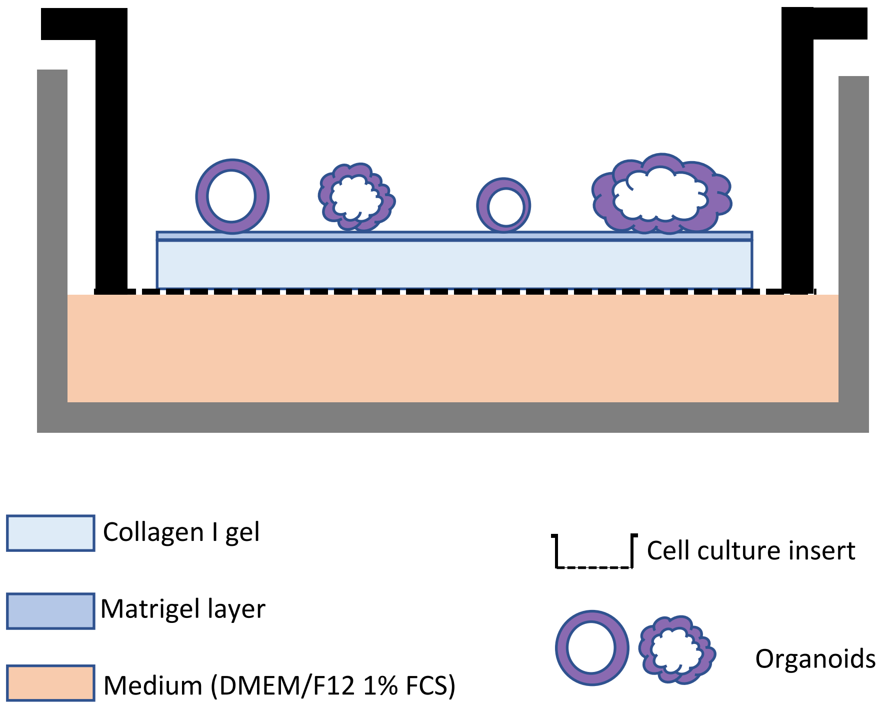

Supplementary Figure S4: Schematic illustration of the experimental setup of organoid culture on top of Matrigel coated collagen gels at the air liquid interface.
